# Virtual reality navigation for the early detection of Alzheimer’s disease

**DOI:** 10.3389/fnagi.2025.1571429

**Published:** 2025-08-20

**Authors:** Sayuri Shima, Reiko Ohdake, Yasuaki Mizutani, Harutsugu Tatebe, Riki Koike, Atsushi Kasai, Epifanio Bagarinao, Kazuya Kawabata, Akihiro Ueda, Mizuki Ito, Junichi Hata, Shinsuke Ishigaki, Junichiro Yoshimoto, Hiroshi Toyama, Takahiko Tokuda, Akihiko Takashima, Hirohisa Watanabe

**Affiliations:** ^1^Department of Neurology, Fujita Health University School of Medicine, Toyoake, Aichi, Japan; ^2^Department of Functional Brain Imaging, Institute for Quantum Medical Science, National Institutes for Quantum Science and Technology, Chiba, Japan; ^3^Laboratory for Alzheimer's Disease, Department of Life Science, Faculty of Science, Gakushuin University, Tokyo, Japan; ^4^MIG (Medical Innovation Group) Inc., Tokyo, Japan; ^5^Department of Integrated Health Sciences, Nagoya University Graduate School of Medicine, Nagoya, Aichi, Japan; ^6^Brain and Mind Research Center, Nagoya University, Nagoya, Aichi, Japan; ^7^International Center for Brain Science, Fujita Health University, Toyoake, Aichi, Japan; ^8^Graduate School of Human Health Sciences, Tokyo Metropolitan University, Tokyo, Japan; ^9^Molecular Neuroscience Research Center, Shiga University of Medical Science, Shiga, Japan; ^10^Department of Biomedical Data Science, Fujita Health University School of Medicine, Toyoake, Aichi, Japan; ^11^Department of Radiology, Fujita Health University School of Medicine, Toyoake, Aichi, Japan

**Keywords:** apolipoprotein E, cognitive dysfunction, entorhinal cortex, glial fibrillary acidic protein, magnetic resonance imaging, spatial memory, P-tau181, virtual reality

## Abstract

**Objective:**

The development of non-invasive clinical diagnostics is paramount for the early detection of Alzheimer’s disease (AD). Neurofibrillary tangles in AD originate from the entorhinal cortex, a cortical memory area that mediates navigation via path integration (PI). Here, we studied correlations between PI errors and levels of a range of AD biomarkers using a 3D virtual reality navigation system to explore PI as a non-invasive surrogate marker for early detection.

**Methods:**

We examined 111 healthy adults for PI using a head-mounted 3D VR system, AD-related plasma biomarkers (GFAP, NfL, Aβ40, Aβ42, and p-tau181), Apolipoprotein E (ApoE) genotype, and demographic and cognitive assessments. Covariance of PI and AD biomarkers was assessed statistically, including tests for multivariate linear regression, logistic regression, and predictor importance ranking using machine learning, to identify predictive relationships for PI errors.

**Results:**

We found significant positive correlations between PI errors with age and plasma GFAP, p-tau181, and NfL levels. Multivariate analysis identified significant correlations of plasma GFAP (*t*-value = 2.16, *p* = 0.0332) and p-tau181 (*t*-value = 2.53, *p* = 0.0128) with PI errors. Predictor importance ranking using machine learning and receiver operating characteristic curves identified plasma p-tau181 as the most significant predictor of PI. ApoE genotype and plasma p-tau181 showed positive and negative PI associations (ApoE: coefficient = 0.650, *p* = 0.037; p-tau181: coefficient = −0.899, *p* = 0.041). EC thickness exhibited negative correlations with age, mean PI errors, and GFAP, NfL, and p-tau181; however, none of these associations remained significant after adjusting for age in linear regression analyses.

**Conclusion:**

These findings suggest that PI quantified by 3D VR navigation systems may be useful as a surrogate diagnostic tool for the detection of early AD pathophysiology. The hierarchical application of 3D VR PI and plasma p-tau181, in particular, may be an effective combinatorial biomarker for early AD neurodegeneration. These findings advance the application of non-invasive diagnostic tools for early testing and monitoring of AD, paving the way for timely therapeutic interventions and improved epidemiological patient outcomes.

## Introduction

Alzheimer’s disease (AD) is a lifespan disorder with symptoms that typically emerge during aging but whose etiology can begin asymptomatically over years to decades. Given this extended time course, the consensus of the medical community is that preventative approaches to clinical diagnosis and care are paramount. Indeed, lifestyle modifications can influence up to 40% of AD risk underscoring the critical need to develop validated clinical protocols for the identification of individuals at early stage risk for AD (González-Madrid et al., 2023). The conventional approach to risk diagnosis is the use of molecular biomarkers to detect early pathological changes before the appearance of clinical symptoms. However, biomarkers are expensive, require invasive procedures, and do not correlate well with current neurocognitive functional and behavioral tests. Thus, there is an urgent need to develop simple non-invasive clinical indicators and to deploy them as surrogate markers for further molecular diagnoses.

In brain pathology, the onset of AD begins in the entorhinal cortex (EC) of the hippocampal formation, marking the initial site for the development of neurofibrillary tangles (NFTs) (Braak and Braak, 1995, 1996). These tangles progressively expand from the EC through the limbic cortex and finally to the neocortex, a process that correlates with the severity of cognitive decline (Jellinger, 2006). As an early indicator of AD, the presence of NFTs in the EC does not directly correlate with dementia symptoms, however, their expansion into limbic cortex and neocortex is commonly associated, respectively, with mild cognitive impairment (MCI) and overt dementia (Jack et al., 2004; Rodrigue and Raz, 2004; Thaker et al., 2017). Thus, the co-occurrence of NFT accumulation with cognitive and behavioral deterioration provides a timeline for clinical brain aging in normal and disease states, with changes in the EC indicating the staged progression from a pre-clinical state to overt clinical deterioration.

The EC contains a grid cell network that performs spatial mapping and navigation capabilities (Hafting et al., 2005) by encoding periodic self-location representations crucial for path integration (PI). PI enables the calculation of one’s current position by continuously updating head orientation and movement over time using self-motion cues from visual, vestibular, and proprioceptive sources, independent of external cues such as landmarks (Segen et al., 2022). In accord, disruptions in grid cell functioning (Gil et al., 2018) or the inhibition of EC cells (Koike et al., 2024) impair PI. Indeed, two types of hippocampal-dependent cognition, PI and spatial memory (SM) have been investigated as behavioral markers for aging using a head-mounted 3D virtual reality (VR) system (Kunz et al., 2015; Stangl et al., 2018; Howett et al., 2019; Koike et al., 2024), an immersive technology that encompasses the user’s field of vision and simulates head and body movements. The 3D VR paradigm indicates that PI errors commence around the age of 50 and increase with age, according to Braak’s classification (Koike et al., 2024). The age-related decline in PI ability suggested the hypothesis that it might serve as a surrogate marker for NFT status in the EC for the detection of early-stage AD.

The combinatorial analysis of EC-dependent PI and conventional AD biomarkers and genetic markers comprise a potentially powerful approach to AD risk diagnosis. Here, we studied the relationships between PI errors assessed using a 3D VR system and a diverse panel of known AD-related and aging-related biomarkers in a cohort of 111 healthy individuals. Our findings reveal a novel combinatorial approach to the use of surrogate behavioral biomarkers on the pathological progression of AD. Although conventional biomarkers such as Aβ42 and phospho-tau can detect underlying amyloid and tau pathology in asymptomatic individuals (Niimi et al., 2024), combining them with sensitive behavioral measures may improve the precision and timing of preclinical AD detection.

## Materials and methods

### Participants

We analyzed data from 140 healthy volunteers that participated in our ongoing aging registry study at Fujita Health University who were recruited between September 2021 and June 2023. Our study was conducted exclusively in Japan, involving a homogeneous population of Japanese participants. We collected demographic data, including education, medical history, medication use, drinking and smoking habits, and family history of neurodegenerative diseases. All participants underwent 3 T magnetic resonance imaging (MRI) and clinical assessments of general cognitive performance using the Mini-Mental State Examination (MMSE) (Folstein et al., 1975), the Japanese version of Addenbrooke’s Cognitive Examination-Revised (ACE-R) (Mioshi et al., 2006), and the Japanese version of the Montreal Cognitive Assessment (MoCA-J) (Nasreddine et al., 2005). The ACE-R is a comprehensive cognitive assessment battery that evaluates multiple domains, including memory, attention, language, and visuospatial skills (Mioshi et al., 2006). A total score of 89 is commonly used as the cutoff normal cognition. Mood disturbances were evaluated using the Geriatric Depression Scale-15 (GDS-15).

Of the 140 initial participants, 29 were excluded because of (a) an inability to complete the 3D navigation task due to VR-induced sickness (9 participants), (b) cognitively abnormal MMSE scores below 26 or ACE-R total scores below 89 (13 participants) (Mizutani et al., 2023), (c) a medical history of stroke (2 participants), or neurological or psychiatric disorders (2 participants), or (d) incomplete data (3 participants). The characteristics of the 111 final participants aged 22 to 79 years are summarized in Table 1. All participants underwent testing on the same day in the following order: blood tests, cognitive assessments, VR-based PI evaluation, and MRI scanning.

**Table 1:** Demographic characteristics of the participants.

Variables	All participants	Mean 3D VR error distance		*p*-value	*APOE* ε4 allele		*p*-value
	*n* = 111	<5 vm, *n* = 72	≥5 vm, *n* = 39		Negative, *n* = 93	Positive, *n* = 18	
Age at examination (range), years	54.8 ± 12.2 (22–79)	52.1 ± 12.2 (22–79)	59.8 ± 10.7 (24–78)	<0.001***	55.8 ± 12.3 (22–79)	49.9 ± 10.9 (24–74)	0.017*
Sex (male/female)	43/68	33/39	10/29	0.037*	39/54	4/14	0.116
Education	14.1 ± 2.3	14.5 ± 2.4	13.5 ± 2.0	0.052	14.3 ± 2.3	13.4 ± 2.1	0.086
MMSE score	29.1 ± 0.9	29.3 ± 0.8	28.9 ± 1.0	0.083	29.1 ± 0.9	29.4 ± 0.8	0.081
ACE-R score	95.5 ± 2.6	95.8 ± 2.3	95.0 ± 2.9	0.277	95.6 ± 2.6	95.2 ± 2.5	0.501
MoCA-J score	26.3 ± 2.0	26.4 ± 2.1	26.1 ± 1.9	0.208	26.2 ± 2.1	26.7 ± 1.9	0.384
GDS-15 score	3.1 ± 2.8	3.2 ± 2.9	2.9 ± 2.5	0.706	3.0 ± 2.7	4.0 ± 3.1	0.157
*APOE* ε4 positivity, *n* (%)	18 (16.2)	9 (12.5)	9 (23.1)	0.149			
Plasma GFAP (pg/mL)	86.21 ± 34.17	77.92 ± 28.20	101.51 ± 39.02	0.001**	87.05 ± 33.27	81.88 ± 39.23	0.648
Plasma Aβ42/Aβ40 ratio	0.07 ± 0.01	0.07 ± 0.01	0.07 ± 0.02	0.208	0.07 ± 0.02	0.07 ± 0.01	0.873
Plasma Aβ42 (pg/mL)	6.15 ± 1.57	6.16 ± 1.39	6.14 ± 1.88	0.587	6.17 ± 1.58	6.05 ± 1.58	0.917
Plasma Aβ40 (pg/mL)	90.36 ± 14.30	89.04 ± 12.45	92.80 ± 17.13	0.231	90.96 ± 13.06	87.28 ± 19.72	0.349
Plasma NfL (pg/mL)	10.27 ± 4.74	9.41 ± 4.02	11.86 ± 5.54	0.014*	10.64 ± 4.54	8.35 ± 5.40	0.028*
Plasma p-tau181 (pg/mL)	1.47 ± 0.61	1.33 ± 0.47	1.72 ± 0.74	0.008*	1.48 ± 0.63	1.39 ± 0.48	0.780

ACE-R, Addenbrooke’s Cognitive Examination-Revised; APOE, apolipoprotein E; Aβ, amyloid β; GDS-15, Geriatric Depression Scale-15; GFAP, glial fibrillary acidic protein; MMSE, Mini-Mental State Examination; MoCA-J, Japanese version of the Montreal Cognitive Assessment; NfL, neurofilament light protein; vm, virtual meter; VR, virtual reality. Means were compared with t tests, and proportions were compared with the *χ*^2^ test. *p < 0.05, **P < 0.01, and ***P < 0.001.

This research was approved by the Ethics Committee at Fujita Health University Hospital (approval number: HM22-407) and conform to the Ethical Guidelines for Medical and Health Research Involving Human Subjects endorsed by the Japanese government. All participants provided written informed consent prior to joining the study and retained the option to withdraw from the study at any point.

### 3D VR navigation task

We developed digital biomarkers for the early detection of AD using head-mounted immersive 3D VR devices. These systems are characterized by their capacity to envelop the user’s field of vision and simulate authentic head or body motions (Koike et al., 2024). The VR space comprised a 20-virtual meter (vm) diameter arena encircled by walls 3 vm in height. Participants wore 3D VR goggles and controlled their movements using a joystick (Meta Quest 2). Initially, they were permitted to move freely within the virtual arena, which included obstacles, to become accustomed to the setup. Forward and backward movements were joystick-controlled, whereas lateral movements required participants to rotate their bodies while seated on a swivel chair.

Prior to testing, participants completed a brief questionnaire to assess motion-sickness susceptibility. Individuals who were unable to reliably operate the joystick or who experienced significant VR-induced discomfort during the calibration phase were excluded. Participants with mild vestibular symptoms received standardized instructions (e.g., smoothly releasing the joystick, keeping their feet still). Each eligible participant then underwent a 2-min calibration procedure to adjust the joystick dead-zone, sensitivity, and headset alignment for individual comfort. Throughout the task, participants remained seated in a swivel chair to minimize postural demands. The entire session, including the practice trial, lasted approximately 15 min and was conducted independently, except in cases where verbal guidance was provided due to hearing difficulties. Volunteers with visual impairments or those unable to effectively use a joystick were excluded. The software and VR goggles described in a previous study (Howett et al., 2019) were provided by MIG Inc. (Tokyo, Japan; https://www.medicalig.com).

**Figure 1:**
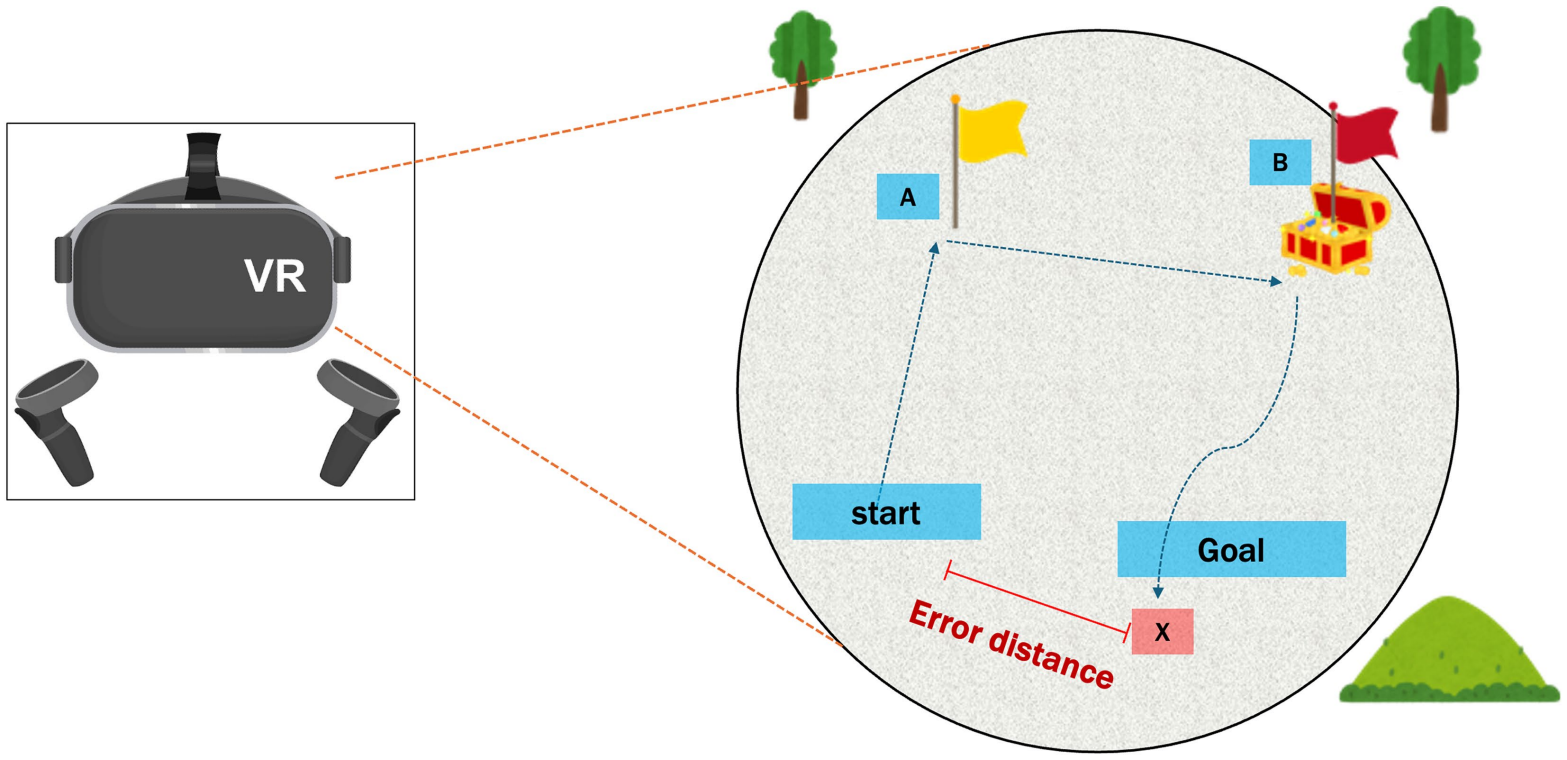
Schematic representation of the 3D VR navigation task. Path integration was measured in VR goggle-wearing participants going to Location A (indicated by a yellow flag), then to Location B (indicated by a red flag), and finally returning to the starting point. The error distance between the participant’s final location (x) and the starting point was determined. VR, virtual reality.

The PI performance was assessed in participants wearing the VR goggles who navigated first to a designated Location A (marked by a yellow flag), proceeded then to a designated Location B (marked by a red flag), and finally returned to the starting point (Figure 1). During the return to the starting point, flag marker at Location A was no longer visible, requiring participants to rely solely on their memory and spatial awareness to navigate back. The distance between the participant’s final position and the actual starting point (error distance) was recorded. The participants completed this test three times, with the average error distance used as the measure of PI ability.

To minimize practice effects, we randomized the starting orientation across three trials and defined our primary PI metric as the mean error over those trials. This three-trial protocol mirrors previously published methods (Hanyu et al., 2024; Koike et al., 2024), which demonstrated stable performance after the first learning exposure.

### Sample collection and assays of plasma biomarkers

Blood plasma was obtained from all recruited participants by collecting blood samples after a fasting period of more than 6 h. The samples were centrifuged for 10 min at 1,500 × g. To prevent repeated freeze–thaw cycles, the obtained plasma was aliquoted into 500 μL samples that were promptly frozen and stored at −80°C until analysis. The plasma levels of plasma glial fibrillary acidic protein (GFAP), neurofilament light (NfL) protein, amyloid β (Aβ)40, Aβ42, and p-tau181 were measured using a single-molecule array (Simoa) with the Simoa Human Neurology 4-Plex A Kit and Simoa pTau-181 V2 Advantage Kit (Quanterix, Billerica, MA, United States), following the manufacturer’s instructions. To ensure technical reliability and account for potential pipetting or assay variability, plasma samples were assayed in duplicate. The enzyme-per-bead values from each pair were averaged to reduce measurement error, and final protein concentrations were determined using a standard curve generated from known calibrator concentrations.

### APOE genotyping

For ApoE genotyping, we used the Invader® assay, as previously described (Arai et al., 2007). Genotyping analysis was performed by calculating the ratio of net fluorescence counts for the wild-type primary probe to that of the mutant primary probe.

### MRI data

All participants underwent brain imaging at Fujita Health University using a 3 T MRI scanner (Canon Medical Systems) with a 32-channel head coil. For each participant, T1-weighted images were acquired using a 3D fast field echo sequence with the following imaging parameters: repetition time = 6.6/2500 ms, echo time = 2.7 ms, flip angle = 8°, field-of-view = 240 × 240 mm^2^, acquisition/reconstruction matrix = 288 × 288, 230 sagittal slices with 0.8-mm slice thickness, and voxel resolution of 0.8 × 0.8 × 0.8 mm^3^, with a total acquisition time of 5 min and 5 s.

All T1-weighted images were preprocessed with Freesurfer v7.2^1^ using its default *recon-all* pipeline. Regional volume and thickness data were then extracted from the preprocessed images using the Desikan-Killiany atlas (Desikan et al., 2006), which consists of 34 cortical regions of interest per hemisphere.

### Statistical analyses

Statistical analyses were conducted to examine the relationship between PI performance and various demographic, clinical, and biomarker measures. We employed the Mann–Whitney U test and the chi-square tests to analyze demographic comparisons between the two independent study groups. Pearson’s correlation analyses of the extracted volume and thickness data were performed using PI and biomarker data; the correlation coefficients and their corresponding *p*-values were computed using the *corr()* function in MATLAB (R2023a; MathWorks, Natick, Mass, United States). We also examined the association between PI and EC thickness and volume using linear regression analysis, which included age, sex, and estimated total intracranial volume (for volume data only) as covariates. To elucidate the factors that significantly predicted PI errors, we performed multivariate linear regression analyses by implementing the standard least squares method. We evaluated the predictive power of age, GFAP, NfL, p-tau181, Aβ40, Aβ42, the ratio of Aβ42/Aβ40 ratio, and *APOE* ε4 allele status. Multicollinearity was assessed by calculating variance inflation factors (VIFs) for all predictors; those with VIF > 10 were removed, and both linear and logistic regression models were re-fitted using the reduced set of variables.

Logistic regression analysis was performed to determine the odds ratios for PI errors exceeding 5 vm based on the same set of predictors. This 5 vm threshold was derived from a previous study employing the same 3D-VR methodology, which showed that participants in their twenties typically exhibited PI errors below 5 vm, whereas the proportion of individuals with PI errors ≥ 5 vm increased progressively with age (Hanyu et al., 2024; Koike et al., 2024). The Wald chi-square test was used to determine the significance of each predictor within the model. To explore a generalizable model for predicting whether PI errors exceed 5 vm based on the above predictors, we trained four supervised machine-learning models: (1) a logistic regression model and (2) a support vector machine with a linear kernel were selected as representatives of linear binary classifiers, (3) a support vector machine with a Gaussian kernel, and (4) a random forest were selected as representatives of non-linear binary classifiers. The generalization performance was then estimated using the leave-one-out cross-validation paradigm. To investigate if the significance of predictors in the model showing the best generalization performance is consistent with those in the logistic regression model, the importance of the predictors was evaluated by a predictor importance ranking method named “model class reliance (MCR)” (Fisher et al., 2019).

Receiver operating characteristic (ROC) curve analysis was used to determine the optimal plasma p-tau181 plasma level cut-off value for predicting significant PI errors. We set the cutoff at 2.2 pg./mL, which is consistent with a previously reported threshold for amyloid-PET and tau-PET positivity using the same Simoa platform (Tagai et al., 2024). The AUC, standard error of the AUC, and 95% confidence interval were computed to assess the accuracy of the p-tau181 plasma level as a predictive biomarker. The sensitivity and specificity of these cut-off values were also calculated to understand the practical applicability of our findings in a clinical setting.

All statistical analyses were performed using JMP software version 16 (SAS Institute, Cary, NC, United States), except that the predictions by supervised machine-learning models and MCR were implemented by a custom-written MATLAB program (version R2024a, MathWorks, Natick, MA, United States). An alpha level of 0.05 was set to determine statistical significance. The hyperparameters of the machine learning model were set to the default values provided by MATLAB without any optimization processes (Specifically, for the support vector machines, the box constraint was set to C = 1, the kernel scale of the Gaussian kernel was set to *γ* = 1. the number of trees was set to 100 for the random forest models).

## Results

Table 1 presents the demographic characteristics of the study groups. The mean age of the entire study population at examination was 54.8 ± 12.2 (range: 22–79) years, and women comprised 61.3%. The 39 (35.1%) participants with a ≥ 5 vm error distance had a higher age at examination, a higher percentage of female participants, and elevated plasma levels of GFAP, NfL, and p-tau181 compared to those with a <5 vm error distance. General cognitive performance assessed by the MMSE, ACE-R, and MoCA-J was not significantly different between the two groups. *APOE* ε4 was positive in 18 (16.2%) participants among the entire study population, and they were younger than those without.

**Figure 2:**
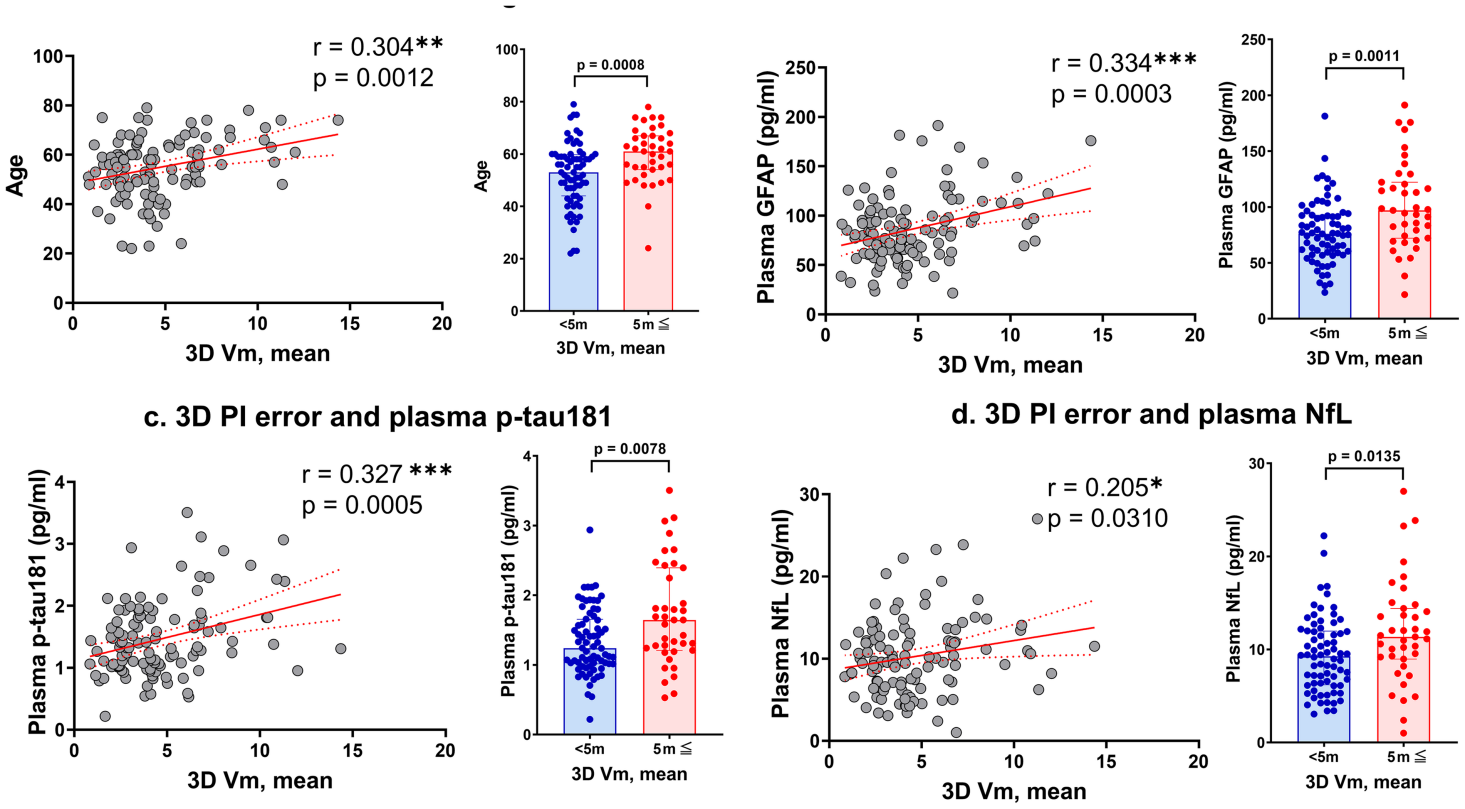
Correlations between 3D VR path integration errors and various markers. Panel **(a)** shows the relationship between mean error distance in the 3D VR navigation task and participant age. Panels **(b–d)** depict the correlations of mean error distance with plasma levels of GFAP, p-tau181, and NfL, respectively. The scatter plots are complemented by bar graphs comparing groups with mean error distances of <5 vm and ≥5 vm. Solid red lines represent regression lines, and red dashed lines show 95% confidence intervals. Significance levels are marked as *p < 0.05, **p < 0.01, and ***p < 0.001. GFAP, glial fibrillary acidic protein; NfL, neurofilament light; vm, virtual meter; PI, path integration; VR, virtual reality.

**Figure 3:**
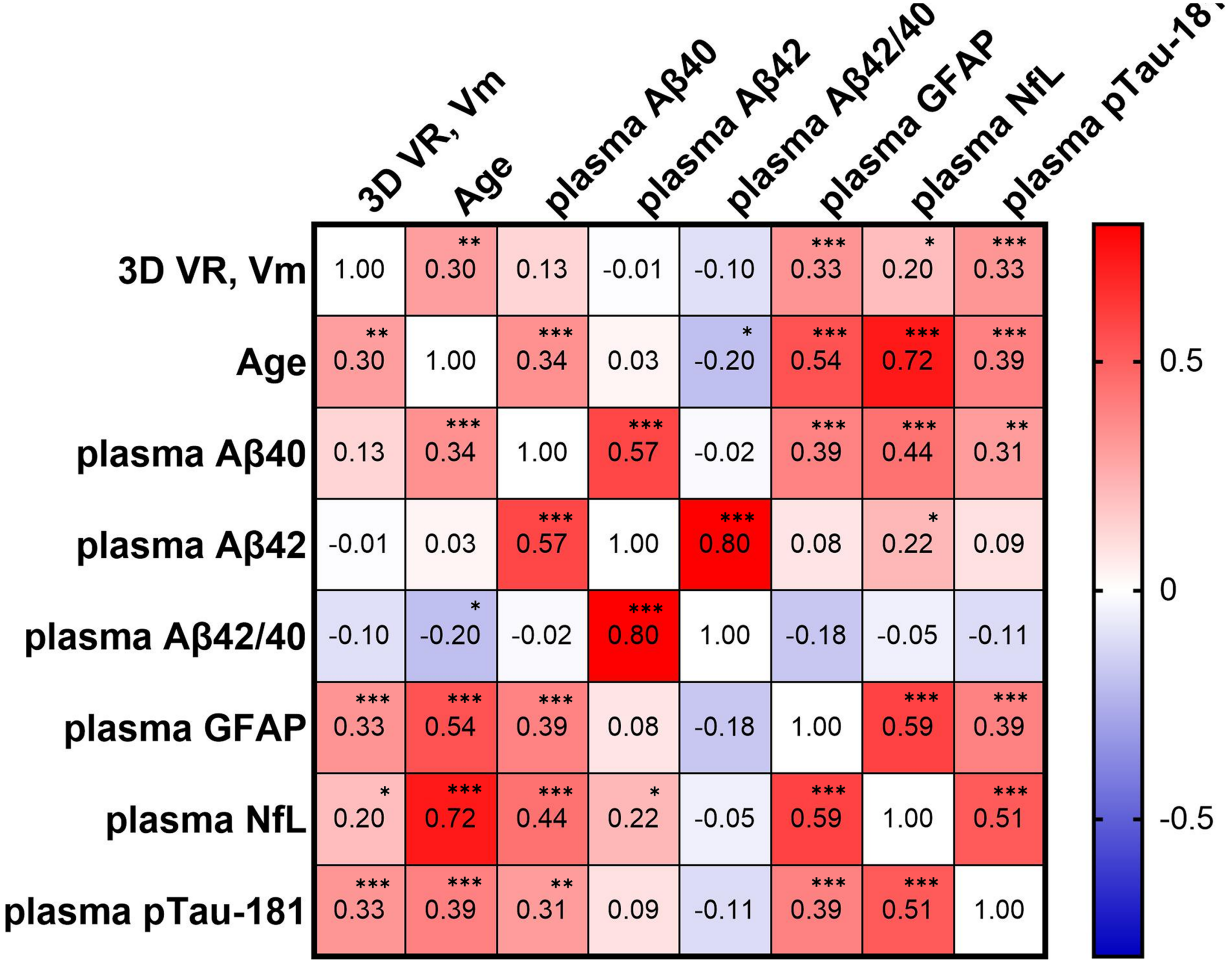
Heatmap depicting the correlation matrix of path integration errors measured by 3D VR navigation (3D VR, vm) and various plasma biomarkers. The matrix presents Pearson’s correlation coefficients between variables including plasma A*β*40, Aβ42, Aβ42/Aβ40 ratio, GFAP, NfL, and p-tau181. Positive correlations are indicated in red and negative correlations in blue, with the color intensity representing the correlation strength. Significant correlations are denoted as *p < 0.05, **p < 0.01, and ***p < 0.001. Aβ, amyloid β; GFAP, glial fibrillary acidic protein; NfL, neurofilament light protein; vm, virtual meter; VR, virtual reality.

PI errors assessed using 3D VR goggles showed significant positive correlations with age (*r* = 0.304, *p* = 0.0012) and plasma levels of GFAP (*r* = 0.334, *p* = 0.0003), p-tau181 (*r* = 0.327, *p* = 0.0005), and NfL (*r* = 0.205, *p* = 0.0310; Figure 2). Notably, no significant correlations were found between PI errors and plasma levels of Aβ40 and Aβ42, as well as the Aβ42/Aβ40 ratio. These findings are visually represented by a comprehensive heatmap that details the strength and direction of relationships between measured biomarkers and PI performance (Figure 3).

Multivariate analyses utilizing standard least squares revealed that plasma GFAP and p-tau181 levels, along with age, were significantly correlated with PI errors (Table 2). In the logistic regression analysis, both plasma p-tau181 levels and the presence of the *APOE* ε4 allele were significantly associated with increased PI errors (Table 3).

**Table 2:** Multivariate analyses utilizing standard least squares for PI errors and potential confounding factors.

Variable	Coefficient	Std. error	*t*-value	*P*-value	95% CI
Interception	−0.203146	1.187987	−0.17	0.8646	(−2.558705, 2.1524121)
Age	0.0563816	0.028327	1.99	0.0491*	(0.0002148, 0.1125485)
Plasma GFAP	0.0191023	0.008772	2.18	0.0317*	(0.0017081, 0.0364965)
Plasma NfL	−0.142004	0.080222	−1.77	0.0796	(−0.301069, 0.0170614)
Plasma p-tau181	1.1607704	0.454761	2.55	0. 0121*	(0.2590626, 2.0624781)
APOE ε4 positivity	−0.164448	0.323482	−0.51	0.6123	(−0.805854, 0.4769566)

APOE, apolipoprotein E; CI, confidence interval; GFAP, glial fibrillary acidic protein; NfL, neurofilament light protein; PI, path integration. *P < 0.05.

**Table 3:** Logistic regression analysis of odds ratios for increased PI errors.

Predictor	Coefficient	Std. error	Wald chi-square	*P*-value	95% CI
Interception	−5.3716217	1.49091	12.98	0.0003*	(−8.5919142, −2.7006078)
Age	0.05815079	0.031003	3.52	0.0607	(0.00004286, 0.12254277)
Plasma GFAP	0.01370331	0.0081967	2.79	0.0946	(−0.0020897, 0.03033886)
Plasma NfL	−0.0612266	0.0749329	0.67	0.4139	(−0.2116501, 0.0853091)
Plasma p-tau181	0.88458064	0.4395787	4.05	0.0442*	(0.04600545, 1.78401044)
APOE ε4 positivity	−0.6526679	0.3112176	4.40	0.0360*	(−1.2851273, −0.0515959)

APOE, apolipoprotein E; CI, confidence interval; GFAP, glial fibrillary acidic protein; NfL, neurofilament light protein; PI, path integration. *P < 0.05.

Increased PI Errors were defined as cases in which PI errors exceed 5 vm.

**Figure 4:**
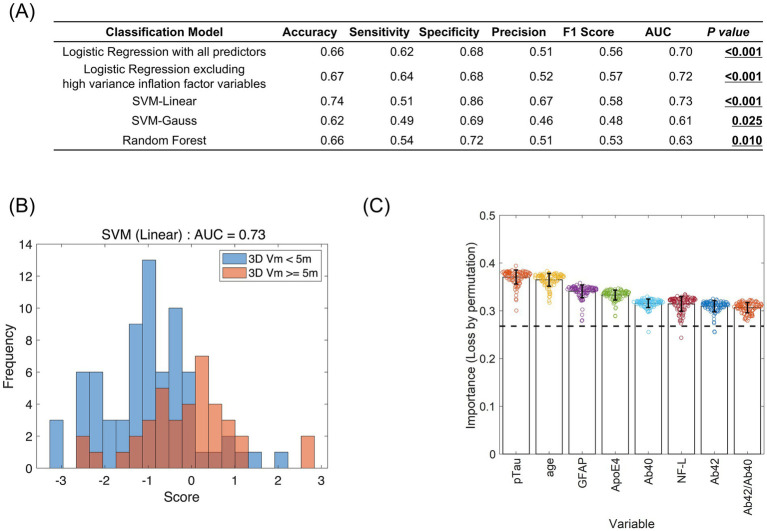
Generalization performance and predictor importance evaluated using machine learning. **(A)** Generalization performance of four machine learning models: Logistic Regression (with all predictors or after excluding high variance inflation factor variables), Support Vector Machine with linear kernel (SVM-linear), Support Vector Machine with Gaussian kernel (SVM-Gaussian), and Random Forest. The first two models were selected as representatives of linear binary classifiers and the last two as representatives of non-linear binary classifiers. The performance measures [accuracy, sensitivity, specificity, precision, F1 score, and AUC (area under the curve of the receiver operator characteristics)] were evaluated using leave-one-out cross-validation (LOOCV). SVM-linear showed the best performance in terms of accuracy and AUC. The *p*-values were evaluated by the permutation test assuming the chance level where the model estimated the classification scores at random. **(B)** Distribution of discriminant scores of the test data in the SVM-linear LOOCV process. The trained model predicted the test data as the positive group (3D vm ≥ 5 m) when the score was 0 or larger and the negative group (3D vm < 5 m) when the score was less than 0. Blue and red denote whether the data actually belong to positive or negative groups, respectively. **(C)** The list of predictor importance scores evaluated by model class reliance (Fisher et al., 2019, Journal of Machine Learning Research 20 (177), 1–81). For each variable, the random permutation was repeated 100 times and the importance scores were evaluated as 1-AUC (higher was more significant) in the LOOCV process. The horizontal dotted line is the 1-AUC achieved by the original (non-permuted) SVM-linear. The bar height and error bars represent the average and standard deviation for 100 random permutations, respectively.

Among the four machine-learning models we explored for predicting whether PI errors exceeded 5 vm, the support vector machine with the linear kernel showed the best accuracy (0.74) and AUC (0.73) evaluated by the leave-one-out cross-validation paradigm (Figures 4A,B). However, the AUC of all models showed significantly better performance than chance, which ranged between 0.61 and 0.73 (Figure 4A). In the support vector machine with the linear kernel, the predictor importance ranking showed that plasma p-tau181 level was the most significant predictor, consistent with the findings from a logistic regression analysis (Figure 4C). Although the *APOE* ε4 allele was recognized as a factor in the logistic regression analysis, its significance was comparatively lower in the support vector machine with the linear kernel model (Figure 4C).

**Figure 5:**
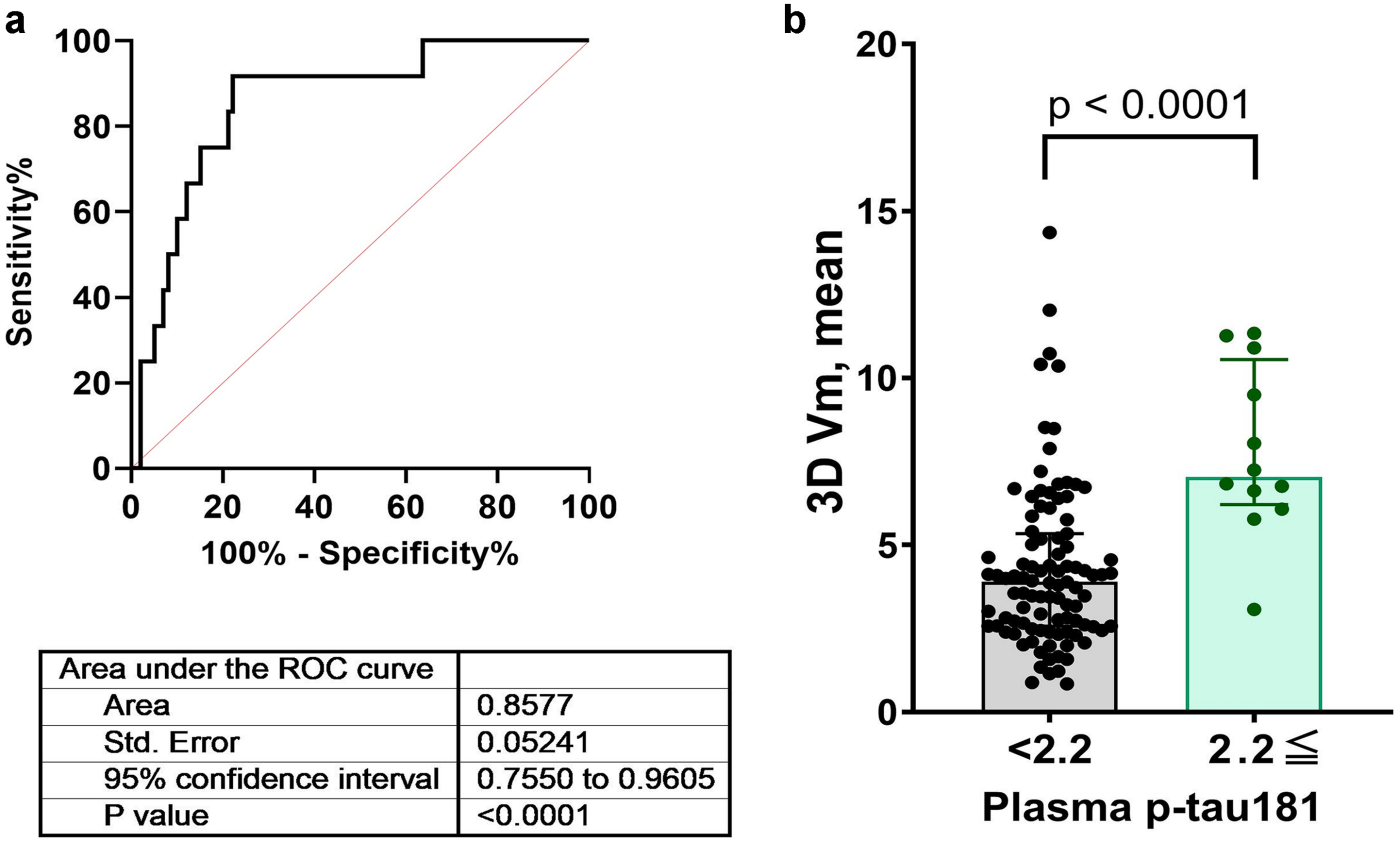
ROC curves assessing the diagnostic performance of plasma p-tau181 levels for detecting path integration errors. Panels **(a,b)** illustrate the ROC curves for a p-tau181 level cutoff of 2.2 pg./mL along with AUC, standard errors, confidence intervals, and *p*-values. AUC, area under the curve; ROC, receiver operating characteristic.

The ROC curve for detecting plasma p-tau181 levels ≥ 2.2 presented an AUC of 0.8577, suggesting a high degree of accuracy in differentiating individuals based on PI errors. The standard error for the AUC was 0.0524, and the 95% confidence interval ranged from 0.7550 to 0.9605, indicating strong model performance. The optimal cut-off value for PI errors was determined to be 5.78 vm, achieving a sensitivity of 91.7% and specificity of 77.8%. The statistical significance of this model was supported by a *p*-value below 0.0001 (Figure 5).

**Figure 6:**
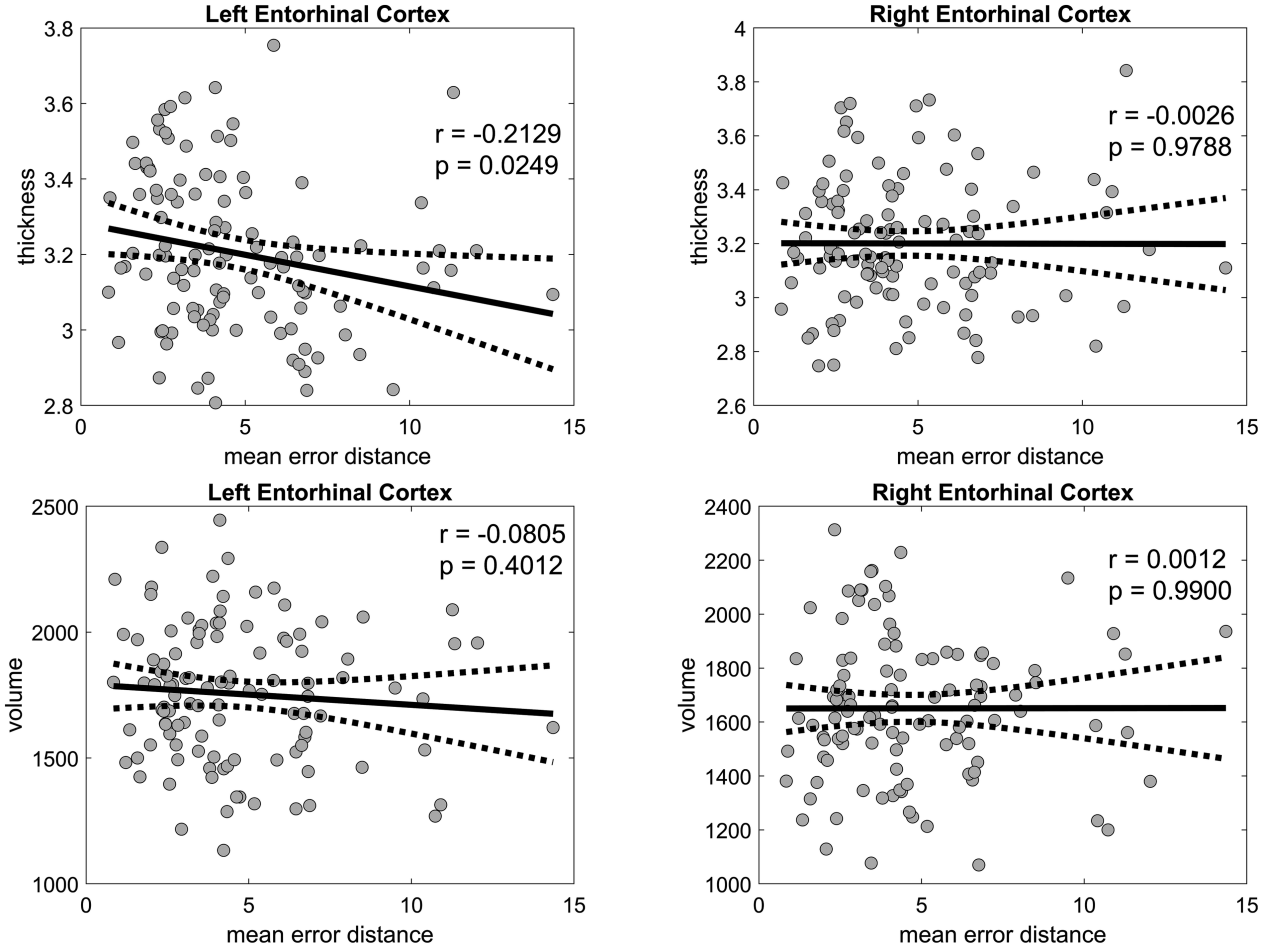
Pearson’s correlation analysis of the entorhinal cortex thickness and volume versus the mean error distance in the 3D VR navigation task. The left entorhinal cortex is shown in the left panels, and the right entorhinal cortex is displayed in the right panels, both with corresponding *p*-values. Solid black lines represent regression fits, and dashed lines represent 95% confidence intervals. VR, virtual reality.

**Figure 7:**
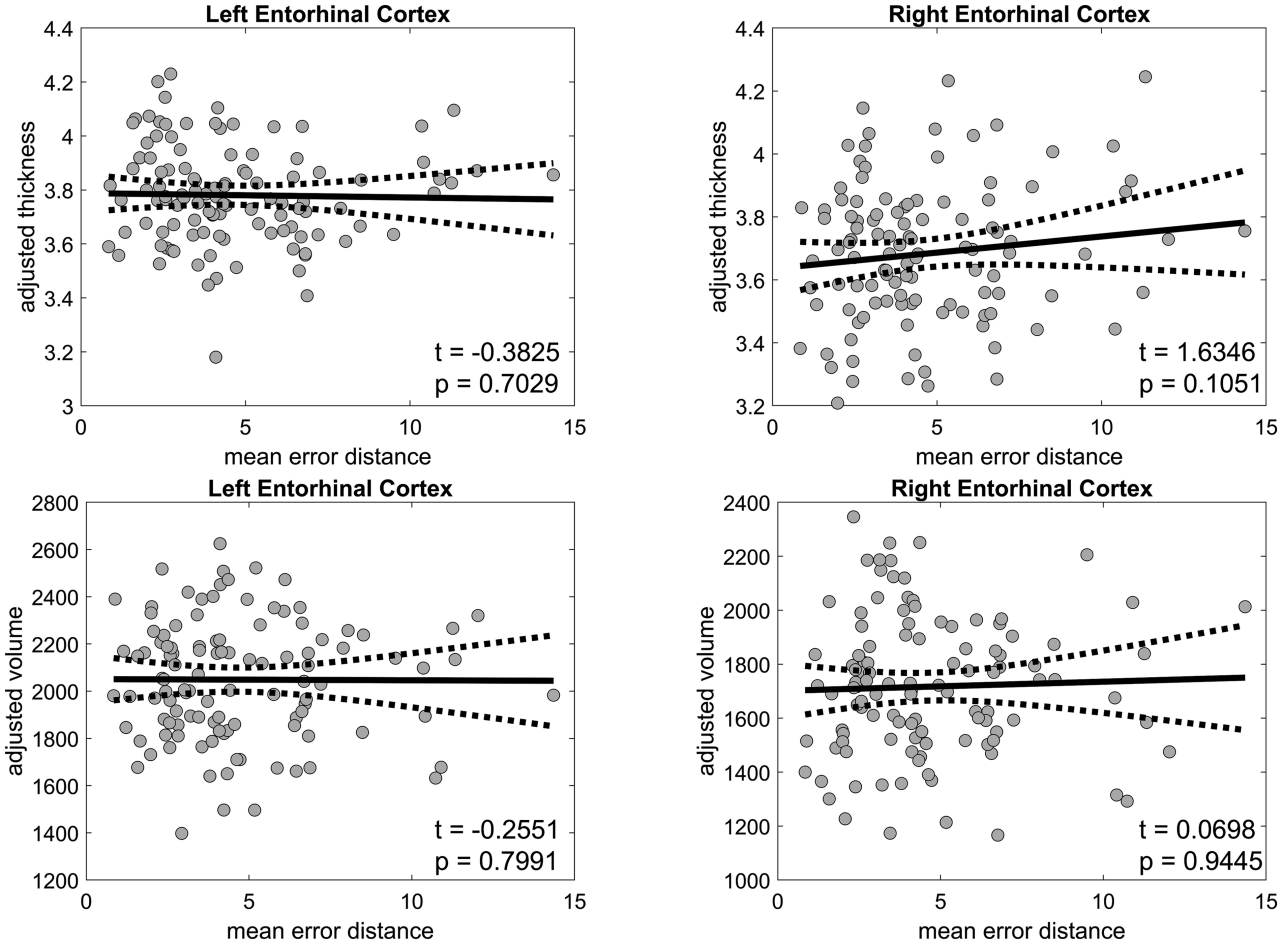
Linear regression analysis of the entorhinal cortex thickness and volume against the mean error distance in the 3D VR navigation task. The left entorhinal cortex is presented in the left panels, and the right entorhinal cortex is shown in the right panels. Statistical significance is denoted where applicable. Solid black lines represent regression fits, and dashed lines represent 95% confidence intervals. VR, virtual reality.

The thickness of the left EC was negatively correlated with age (*r* = −0.4310, *p* < 0.001) and mean PI error (*r* = −0.2129, *p* = 0.0249) (Figure 6), as well as with several biomarkers, including Aβ40 (*r* = −0.2011, *p* = 0.0343), GFAP (*r* = −0.1971, *p* = 0.0381), NfL (*r* = −0.2328, *p* = 0.0139), and p-tau181 (*r* = −0.2373, *p* = 0.0122) levels. Likewise, the thickness of the right EC was negatively correlated with age (*r* = −0.3628, *p* < 0.001), Aβ40 level (*r* = −0.2242, *p* = 0.0180), and NfL level (*r* = −0.2560, *p* = 0.0067). In the linear regression, which included age and sex as covariates, the association between the thickness of the right and left EC and PI error was not significant (Figure 7). No significant associations were observed between EC volume and these measures.

## Discussion

In this cohort of 111 cognitively normal adults, we demonstrate that errors in an entorhinal-dependent 3D VR PI task correlate with plasma biomarkers of neurodegeneration, notably GFAP and p-tau181. In multivariate models adjusting for age and other covariates, both GFAP (*t* = 2.16; *p* = 0.033) and p-tau181 (*t* = 2.53; *p* = 0.013) remained independent predictors of PI error, and machine-learning feature ranking consistently identified p-tau181 as the strongest predictor. Although these findings suggest that PI performance may reflect early AD-related neurobiology, longitudinal studies linking PI deficits to clinical conversion are necessary before PI can be definitively regarded as a predictor of AD risk.

Recent longitudinal and biomarker-driven studies have demonstrated that entorhinal-based PI deficits emerge well before clinical symptoms of AD. Howett et al. showed that PI errors in immersive VR differentiate CSF biomarker–positive MCI patients from controls with an AUC of 0.90 (Howett et al., 2019). Castegnaro et al. (2023) found that overestimation in angular PI and increased variability in PI trajectories are early markers of impending Alzheimer’s dementia. Hanyu et al. (2024) reported that impaired PI performance predicted 12-month cognitive decline in prodromal AD subjects. Most compellingly, Newton et al. demonstrated in a 3-year follow-up of 100 asymptomatic middle-aged adults that each 1 m increase in baseline PI location error in 3D VR was associated with a 2.4-fold higher risk of conversion to MCI (HR 2.4; 95% CI 1.3–4.2; *p* = 0.005), independent of APOE ε4 genotype and education (Newton et al., 2024). Collectively, these data suggest that PI error may represent a sensitive early behavioral biomarker of AD pathology. We therefore propose that future longitudinal analyses in our aging registry combine PI errors with plasma p-tau181 levels to maximize early detection of individuals at imminent risk for clinical conversion.

Our analysis revealed that PI errors in the 3D VR navigation system were significantly correlated with older age and elevated plasma levels of AD-related biomarkers including GFAP, p-tau181, and NfL. A multivariate analysis suggested that plasma GFAP level, APOE ε4 positivity, and, especially, plasma p-tau181 level are more significant predictors of PI performance than age. Further machine learning–based analysis confirmed that plasma p-tau181 was the single strongest predictor of PI error, outperforming GFAP, NfL, APOE ε4 status, and age. These findings indicate that the observed PI errors are not solely a consequence of aging but may also reflect an underlying AD pathology. Moreover, our results highlight the utility of the 3D VR navigation system in capturing these deficits, suggesting its effectiveness as a potential surrogate marker for early pathophysiological changes indicative of AD.

Recent advancements have highlighted the potential of plasma p-tau181 levels as a non-invasive biomarker for AD (Janelidze et al., 2020; Palmqvist et al., 2021), although it is unclear whether the observed p-tau181 levels reflect tau deposition in the EC because plasma p-tau181 levels are not only related to tau positron emission tomography (PET) but also to amyloid PET. This biomarker not only predicts disease progression but also acts as an early pathological marker, with elevated levels correlating with the initial neuropathological changes (Smirnov et al., 2022). The current study found a significant relationship between PI errors and plasma p-tau181 levels, suggesting that these measurements may be efficacious in detecting the progression of AD pathology apart from deficits associated with normal aging. Moreover, ROC analyses showed that PI errors can predict increased plasma levels of p-tau181. These findings suggest that combining VR-based PI metrics with plasma p-tau181 could potentially improve the discrimination of normal age-related navigational changes from those related to early AD pathology. Future work incorporating gold-standard clinical diagnoses and longitudinal follow-up will be essential to confirm these preliminary observations.

Nonetheless, plasma p-tau181 is not entirely specific to AD; elevated levels have also been reported in dementia with Lewy bodies (Gonzalez et al., 2022), Parkinson’s disease with dementia (Mizutani et al., 2023), and amyotrophic lateral sclerosis (Vacchiano et al., 2023). In contrast, plasma p-tau217 and p-tau231 exhibit greater dynamic range and higher specificity for AD pathology in both CSF and blood (Teunissen et al., 2025). Additionally, the [^18^F]MK-6240 tau PET tracer detects early tau accumulation in the entorhinal cortex following amyloid positivity (Cody et al., 2024), reflecting a later stage of tau spread relative to plasma p-tau biomarkers. Combining plasma p-tau181, p-tau231, and [^18^F]MK-6240 PET measures in future studies may therefore help elucidate the temporal sequence of tau pathology and clarify the mechanistic relationship between entorhinal dysfunction and PI errors in our 3D VR navigation task.

Based on univariate analyses, the thickness of the EC negatively correlated with age and mean PI errors, and several biomarkers, but these associations were not statistically significant after adjusting for age and other confounding variables, consistent with a previous report (Newton et al., 2024). Interestingly, EC thickness was correlated with PI ability in people with MCI (Howett et al., 2019) indicating the functional impairment of the EC before significant structural changes are detectable, and that EC thickness changes may parallel the progression of navigational and cognitive decline in AD;^5^ they may be independent predictors when controlling for age.

The PI correlated with plasma GFAP levels but not with the plasma Aβ42/Aβ40 ratio, which has lower sensitivity than the corresponding ratio in cerebrospinal fluid and amyloid PET data (Janelidze et al., 2021). GFAP, an astrocytic cytoskeletal protein of which blood levels are increased in individuals with AD and MCI, is upregulated around Aβ plaques and correlates with tau accumulation, indicating its involvement in neuroinflammatory responses and astrocytic reactivity (Kim et al., 2023). The lack of correlation between the plasma Aβ42/Aβ40 ratio and PI ability might not be caused solely by the low sensitivity of the plasma Aβ42/Aβ40 ratio. The presented correlation between plasma GFAP and PI performance, independent of the plasma Aβ42/Aβ40 ratio, suggests the potential of GFAP as a reactive marker for neuroinflammation. The lack of association with the Aβ42/Aβ40 ratio could stem from multiple factors, including the inherently lower sensitivity of plasma Aβ measures compared to those of cerebrospinal fluid or PET imaging, the specificity of GFAP as a neuroinflammatory marker, the timing of its elevation in the disease course, and the possible disconnect between soluble biomarkers and plaque deposition.

Our findings contribute to advancing the understanding of AD pathophysiology, support the development of non-invasive diagnostic approaches for early detection, and offer a potential clinical framework for implementing preventative interventions at preclinical stages. Continued research is warranted to clarify the specific roles and temporal dynamics of individual biomarkers and to elucidate how they interact within the broader context of AD progression.

### Limitations

Our study had several limitations that warrant consideration. First, our cohort was composed exclusively of Japanese adults residing in predominantly urban environments, specifically Toyoake City and the greater Nagoya area. Their navigational experiences and spatial strategies may have been shaped by the local city layout and cultural factors, potentially limiting the generalizability of our findings to populations with different environmental exposures. Previous work by Coutrot et al. (2022) has shown that individuals raised in more complex, less grid-like street networks—often found in rural or organically developed areas—tend to develop superior wayfinding abilities. These findings suggest that lifestyle, environmental familiarity, and cultural context may modulate PI performance. To validate the universality of PI error as an early behavioral biomarker of AD, future studies should include participants from diverse geographic, cultural, and environmental backgrounds.

Second, while our 3D VR navigation system has demonstrated potential for detecting early PI deficits, it does not evaluate contributing sensory information, such as visual flow, vestibular function, and proprioception, which are integrated within the EC and decline with age. This limitation suggests that our findings may reflect not only early markers of AD but also normal age-related changes, making it a challenge to distinguish between the two.

Third, we applied a single PI error cutoff (>5 vm) across the 20–85 year span. This threshold was originally defined in our Brain Communications report (Koike et al., 2024) based on the 95% upper confidence bound of error distances in a tightly clustered 20s cohort. However, participants aged ≥ 50 years display both a broader dispersion and a rightward shift in error distances relative to 20s adults, so a uniform cutoff risks conflating normal, age-related navigational decline with early AD-related deficits. Although all multivariate models adjusted for age as a continuous covariate—and our proof-of-concept analysis still demonstrated a robust PI–p-tau181 association—residual age-related confounding cannot be excluded. To better disentangle healthy aging from pathology, future studies should (1) establish decade-based 95th-percentile PI thresholds or calculate age-normed PI *z*-scores; and/or (2) explicitly model age × PI-error interaction effects when evaluating PI performance as an early AD biomarker.

Fourth, we did not apply formal corrections for multiple comparisons in our secondary multivariate analyses. Although our primary hypotheses were pre-specified, the exploratory nature of these additional tests may increase the risk of false-positive findings. In future studies, we plan to address this issue by applying false-discovery-rate or family-wise-error-rate corrections, limiting the number of tested outcomes, and/or utilizing hierarchical modeling approaches. Validation in independent cohorts will also be necessary to confirm the robustness of our findings.

Finally, the absence of detectable changes in AD-related plasma biomarkers in participants with PI errors highlights the need for a broader range of diagnostic modalities to fully assess the early stages of AD pathophysiology. Furthermore, the imbalance in sample size for participants with the APOE ε4 allele might affect the generalizability of our results. Future studies incorporating sensory evaluations with larger and more diverse cohorts are essential to validate our findings and refine the utility of VR systems in early AD detection.

## Data Availability

The raw data supporting the conclusions of this article will be made available by the authors, without undue reservation.

## Footnotes

^1^ https://surfer.nmr.mgh.harvard.edu/
